# Curbing Inflammation in the Ischemic Heart Disease

**DOI:** 10.1155/2013/183061

**Published:** 2013-05-30

**Authors:** Paulo Roberto B. Evora, Julio Nather, Paulo Victor Tubino, Agnes Afrodite S. Albuquerque, Andrea Carla Celotto, Alfredo J. Rodrigues

**Affiliations:** Department of Surgery and Anatomy, Ribeirão Preto Faculty of Medicine, University of São Paulo, Avenida Bandeirantes 3900, 14048-900 Ribeirão Preto, SP, Brazil

## Abstract

A modern concept considers acute coronary syndrome as an autoinflammatory disorder. From the onset to the healing stage, an endless inflammation has been presented with complex, multiple cross-talk mechanisms at the molecular, cellular, and organ levels. Inflammatory response following acute myocardial infarction has been well documented since the 1940s and 1950s, including increased erythrocyte sedimentation rate, the C-reactive protein analysis, and the determination of serum complement. It is surprising to note, based on a wide literature overview including the following 30 years (decades of 1960, 1970, and 1980), that the inflammatory acute myocardium infarction lost its focus, virtually disappearing from the literature reports. The reversal of this historical process occurs in the 1990s with the explosion of studies involving cytokines. Considering the importance of inflammation in the pathophysiology of ischemic heart disease, the aim of this paper is to present a conceptual overview in order to explore the possibility of curbing this inflammatory process.

## 1. Introduction

Inflammatory response following acute myocardial infarction (AMI) has been documented since the 1940s and 1950s, including increased erythrocyte sedimentation rate (ESR), the C-reactive protein analysis (CRP), and the determination of serum complement (C′). Boltax and Fischel (1956) using serial assay of the ESR, C′, and CRP in sixty-one AMI episodes observed that such tests were positive in over 90% of patients by the third day from the onset of the disease [1].

**Figure 1:**
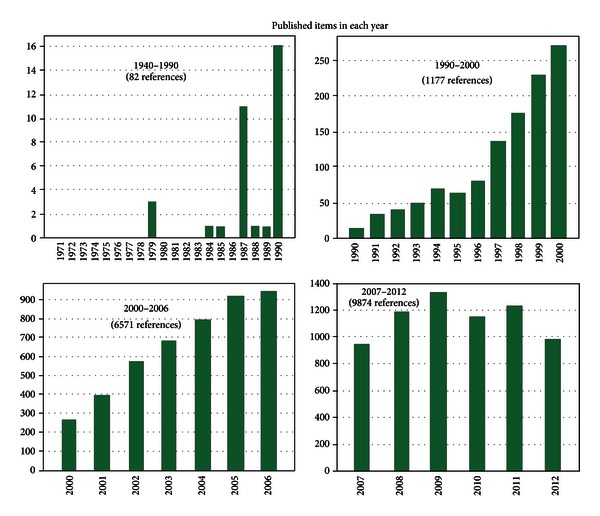
Web of Science timespan references (1940–2012).

In 1943, Lofstrom reported that patients with myocardial infarction also presented the “non-specific capsular swelling in pneumococci,” later associated with the presence of the “C-reactive protein” [2]. Since then, a number of studies have confirmed the occurrence of CRP in myocardial infarction and other noninfectious inflammatory conditions [3, 4]. Surprisingly, an extensive literature overview including publications from 1960s to the 1980s revealed that the role of the inflammation in the AMI lost relevance, virtually disappearing from the literature reports. The reversal of this historical process occurred in the 1990s with the upsurge of investigations involving cytokines (Figure 1).

Therefore, considering the importance of inflammation in the pathophysiology of ischemic heart disease (IHD), the aim of this review is to present an overview of concepts in order to explore the possibilities for curbing the inflammatory process associated with myocardial infarction.

## 2. Inflammation and Ischemic Heart Disease

Nowadays acute coronary syndrome (ACS) has been considered an autoinflammatory disorder comprising the molecular, cellular, and organ multiple cross-talk mechanisms. Even though, early reperfusion, either by thrombolysis or percutaneous coronary intervention, provides excellent clinical benefits in patients with ACS, the ischemia/reperfusion injury may somewhat offset those positive advantages. Although being potentially protective, inflammation has been associated with potentially detrimental conditions such as activation of leukocytes, endothelial cells, vascular smooth muscle cells, platelets, and oxidative stress [5].

Therefore, the inflammation in response of ischemia and necrosis of cardiac tissue has a crucial role not only in tissue repair but also in the prognosis of patients. Biasucci and colleagues (2000) summarized the current concepts of the inflammatory reaction associated with coronary artery disease (CAD). In patients with unstable angina, coronary atherosclerotic plaques are characterized by the presence of macrophages, and to a lesser extent, T-lymphocytes, at the immediate site of either plaque rupture or superficial erosion. Moreover, the rupture-related inflammatory cells are activated, indicating ongoing inflammation at the site of plaque disruption. These observations corroborate the results of clinical studies demonstrating activated circulating neutrophils, lymphocytes, and monocytes, increased concentrations of proinflammatory cytokines, such as interleukin (IL) 1 and 6, and acute phase reactants in patients with unstable angina and myocardial infarction. High levels of C-reactive protein have been associated with an increased risk of in-hospital and later new coronary events in patients with unstable angina, as well as with increased long term risk of death and myocardial infarction in apparently normal subjects. Hence, the cumulative evidences suggest that inflammation may cause local endothelial activation and plaque fissure resulting in unstable angina and myocardial infarction. Although no information is available about why, when, and where exactly the inflammatory process begins, these concepts stimulate researches that may lead to a different approach to the patients with acute coronary syndromes [6].

Two different inflammatory processes take place in patients experiencing AMI. One is in the coronary arterial inflammation that results in AMI and the other occurs in the myocardial and leads to ventricular remodeling. These processes are positively and negatively regulated by Th1 and Th2 lymphocytes, respectively. In an investigation to clarify whether the T-helper (Th)1/Th2 imbalance is involved only in the coronary arteries inflammation or also in the myocardial inflammation and also to explore the importance of the imbalance of Th1/Th2 in the AMI, Cheng and colleagues (2005) observed that IFN-gamma-producing T cells were significantly increased in patients with AMI and unstable angina within 24 hours after the onset of symptoms. They also observed that the high ratio of IFN-gamma-producing T cells had normalized 1 week after the recovering of an unstable angina episode but was still observable 1 week and even 1 month after the AMI. The upregulation of Th1 cell function is compatible with a diseased heart function. There was no significant difference in the frequencies of IL-4-producing T cells 1 week, 2 weeks, and 1 month after AMI. IFN-gamma mRNA increased in the myocardium of rats, but there was no significant change in global Th cell functions. The conclusions were (1) Th1/Th2 functional imbalance exists in both coronary arterial inflammation and myocardial inflammation processes and (2) the upregulation of Th1 cell functions may participate in the immune-mediated ventricular remodeling after AMI [7].

## 3. Acute Myocardial Infarction and Systemic Inflammatory Response

Cardiogenic shock is a devastating consequence of AMI associated with extremely high mortality. The treatment focuses on improving myocardial perfusion/reperfusion and hemodynamic support. Therefore, the main approach is an emergency angiography followed by coronary revascularization by percutaneous intervention or coronary artery bypass grafting. Circulatory support using diastolic intra-aortic balloon pump is frequently used in association with the pharmacological support with vasoactive and inotropic drugs, even though their benefit on survival has not been shown [8]. 

Recent lines of evidence suggest that systemic inflammatory response, including iNOS upregulation, complement activation, and the cascade of inflammatory cytokines, have a role in the development of cardiogenic shock. Therefore, new strategies to restrain the inflammatory process, including the use of C5 and NOS inhibitors, would be combined to the traditional strategies to treat cardiogenic shock.

Since the systemic inflammatory response (SIRS), complement activation, release of inflammatory cytokines, expression of inducible NO synthase (iNOS), endothelial activation, and inappropriate vasodilatation play a critical role in the genesis as well as in the evolution of the cardiogenic shock, new interpretations and therapeutic strategies have been evolved to deal with this ominous consequence of the AMI, as exposed by Reynolds and Hochman (2008) [9]. 

The tilarginine, an LN-monomethyl arginine (L-NMMA) or N(G)-monomethyl-L- arginine HCL, is a nonselective inhibitor of nitric oxide synthase (NOS), which has been studied for treating septic shock and cardiogenic shock complicating myocardial infarction. There is evidence that overproduction of nitric oxide (NO) may contribute to the pathogenesis of cardiogenic shock after myocardial infarction, which is similar to the observed in septic shock. The results of investigations using NOS inhibition in those two disorders have proved disappointing. However, the use of an inducible NOS inhibitor for reducing the pathological effects of excessive NO production might be useful [10, 11]. 

However, the results of experimental researches in animals as well as in humans have been promising. However, investigations in humans (TRIUMPH) with a larger sample whose objective was to assess tilarginine have recently been terminated due to the lack of efficacy and the tendency to increased mortality. The unfavorable evidence of the iNOS inhibition in cardiogenic shock resulted in considerable challenging: “The tragedy of TRIUMPH inhibition of nitric oxide synthesis: where do we go from here” [12, 13].

Methylene blue (MB), a guanylate cyclase inhibitor, can abolish the relaxation of vascular smooth muscle cyclic GMP-dependent without interfering with the NO synthesis and tissue necrosis associated with the use of NOS inhibitors. Therefore, MB may be a therapeutic option, untested, for vasoplegia associated with cardiogenic shock [14, 15] 

## 4. Biomarkers

Since myocardial infarction onset is usually easily timed, it is possible to evaluate the effectiveness of biomarkers in the course of the AMI [1]. Therefore, there have been line of evidence suggesting that new biomarkers combined with cardiospecific troponin, CPR and ERS, may increase the sensitivity of diagnosing acute coronary syndrome [16].

Atherosclerosis is an inflammatory disease, and increased blood levels of inflammatory biomarkers have been observed in acute coronary syndromes. In addition, high expression of inflammatory markers is associated with a worse CAD prognosis. Thus, the most frequent biomarkers used in humans and animal investigations are (1) plasma levels of cytokines IL-6, IL-8, and TNF-*α*; (2) membrane expression of Toll-like receptors 2 and 4; (3) CD11b, CD62L, and CD14 on monocytes and granulocytes as markers of inflammation [17].

Elevated CRP levels have been associated with serious adverse cardiac events including death. However, the causal association of CRP with atherogenesis is less clear, and there are data suggesting that it is a bystander rather than a true risk factor. Importantly, CRP levels decrease in response to anti-inflammatory agents, making it useful for monitoring the efficacy of novel anti-inflammatory drugs [18]. The ESR and CPR analyses are the oldest markers of AMI and are still useful on the clinical practice.

## 5. Curbing Inflammation

According to Klingenberg and Luscher (2012), there are several promising anti-inflammatory drugs that have been tested, and four aspects appear to be paramount for interpreting the results of future trials. First, an anti-inflammatory agent should interfere with inflammatory pathways known to be crucially involved in the pathogenesis of atherosclerosis, but unlike statins such anti-inflammatory agent should attenuate inflammation *per se *and not interfere with lipid levels or other risk factors. Second, a biomarker which reflects the activity of the inflammatory pathway would be required for monitoring the treatment. Third, appropriate identification of patients likely to benefit from this treatment is essential. Either individuals at high risk for cardiovascular events identified by traditional risk scores or patients at high risk for recurrent events after AMI may be considered proper candidates. Fourth, choosing an adequate time point within the natural course of atherosclerosis and the duration of therapy are vital considerations. Obviously, an anti-inflammatory therapy would only provide real clinical benefit if its effectiveness is beyond that of existing usual therapies and cost effective [18].

A number of experimental and clinical investigations have highlighted the key role of inflammation in all phases of atherosclerosis, from fatty streaks to disrupted plaques. Higher levels of inflammatory markers have been associated with poor outcome despite the optimal treatment, including myocardial revascularization. In a thorough review Bona and colleagues focused on inflammation as a potential new therapeutic target of ACS appraising four anti-inflammatory treatments: (1) nonspecific anti-inflammatory drugs; (2) specific antagonists of key cytokines; (3) immunomodulatory therapies; (4) immunization as promising therapy against atherosclerosis [19]. Klingenberg and Luscher (2012) have published another worthy review [18], and both reviews are essential for those interested in potential therapeutic strategies for “curbing inflammation.”

The early inflammatory process, the innate inflammation, would be a protective reaction in the acute phase of myocardial infarction. However, the overtime inflammatory response should be curbed. Therefore, based on the concepts of ischemic myocardial protection established in the 1970s, it would be inappropriate to curb inflammation within 6 hours after the onset of AMI. However, according to Timmers and colleagues (2012) translation of therapeutic anti-inflammatory strategies to reduce myocardial ischemia/reperfusion injury into clinical practice appears to be a challenging task since general inhibition of the innate immune system is associated with adverse outcomes after myocardial infarction. The challenge is to inhibit those parts of the innate immune system that cause injury without affecting the myocardial infarct healing. The current body of knowledge is limited to understand the spatial and temporal functions of endogenous ligands and their receptors, inflammatory cells, and inflammatory mediators with pleiotropic and synergistic or antagonistic effects in myocardial ischemia/reperfusion injury [20].

The natural history demonstrated that early reperfusion (thrombolysis, PTCA, and surgery) has a positive impact on the AMI evolution, resulting in a significant reduction of cardiogenic shock, ventricular aneurysms, and death. Thus, this presents a further question: should all patients undergo anti-inflammatory treatment or only those that are experiencing elevated levels of biomarkers, especially CRP, ESR, and complement? In addition, based on the variety of individual clinical evolution after AMI (cardiogenic shock, progression to dilated cardiomyopathy, ventricular aneurysms, and SIRS), the involvement of genetic factors is clear. Thus, ascertain the genetic predisposition in conjunction with the presence of biomarkers of inflammation should be an initial step for curbing inflammation associated with AMI. 

Finally, two hypothetical questions have to be addressed. It is well known that inflammation occurs in the wall of the coronary arteries, atherosclerotic plaque, and myocardium, raising the question if these processes should be considered individually or as part of a unique process of inflammation. In addition, one should consider if conventional medications (ACE inhibitors, statins, aspirin, nitrates, and beta-blockers) would be no longer functioning as curbing the inflammatory process associated with AMI? 

Table 1 summarizes the physiopathological “key points”, and “key points” for curbing inflammation in ischemic heart disease are summarized in Table 2.

## Figures and Tables

**Table 1:** Ischemic heart disease and inflammation—key physiopathology concepts.

(i) A modern concept considers ACS as an autoinflammatory disorder.	
(ii) Inflammatory response following AMI has been well documented since the 1940s and 1950s.	
(iii) It is surprising to note, based on extensive literature overview including the following 30 years (decades of 1960, 1970, and 1980), that the inflammatory AMI lost its focus, virtually disappearing from the literature reports.	
(iv) There are two different inflammatory processes in patients with AMI: the coronary arterial inflammation that leads to the pathogenesis of AMI, followed by myocardial inflammation that leads to ventricular remodeling.	
(v) Systemic inflammatory response (SIRS), complement activation, release of inflammatory cytokines, iNOS expression, and vasodilatation cannot only play a pivotal role in the genesis and evolution of shock.	
(vi) The most frequent biomarkers used in humans and experimental protocols are (1) plasma levels of cytokines IL-6, IL-8, and TNF-*α*; (2) membrane expression of Toll-like receptor; (3) CD11b, CD62L, and CD14 on monocytes and granulocytes as markers of inflammation.	
(vii) Curiously, increased erythrocyte sedimentation rate (ESR) and the C-reactive protein analysis (CRP) are the oldest markers of AMI and still are the most useful on the clinical practice.	

**Table 2:** Curbing inflammation in ischemic heart disease—key points.

(i) An anti-inflammatory therapy would provide real clinical value if an incremental benefit above and beyond existing therapies in a cost-efficient approach could be provided.	
(ii) A potential new therapeutic target of ACS includes at least four anti-inflammatory treatment options: (1) nonspecific anti-inflammatory drugs; (2) specific antagonists of key cytokines; (3) immunomodulatory therapies; (4) immunization as promising therapeutic modality against atherosclerosis.	
(iii) There is an early inflammatory response (innate inflammation) that would be a protective reaction in the acute phase of MI. Over time, persisting inflammatory response should be curbed.	
(iv) The onset of AMI is determined with a certain safety margin. Thus, based on the concepts of ischemic myocardial protection emanating from the 1970s, it would be inappropriate “curbing” inflammation within 6 hours.	
(v) General inhibition of the innate immune system is associated with adverse outcome after the challenge being to inhibit those parts of the innate immune system that cause injury, without affecting the myocardial infarct healing.	
(vi) Would the sense of genetic predisposition, based on sensitive biomarkers, be an initial step to get strategies for AMI curbing inflammation?	
(vii) It is well known that the inflammation occurs in the coronary artery wall, in the atherosclerotic plaque, and the myocardium. Would these alterations be considered individually or as a part of a single process of inflammation?	
(viii) Would regular medications (ACE inhibitors, statins, aspirin, nitrates, and beta-blockers) be no longer functioning as curbing the AMI inflammatory process?	
